# Camel Milk Protein Ameliorates Ulcerative Colitis by Modulating Gut Microbiota and Amino Acid Metabolism

**DOI:** 10.3390/nu17050780

**Published:** 2025-02-24

**Authors:** Ning Kang, Zhexin Fan, Li Yang, Jie Shen, Yuechenfei Shen, Zhifeng Fang, Baokun Li, Bo Yang, Jiancheng Wang

**Affiliations:** 1 Key Laboratory of Agricultural Product Processing and Quality Control of Specialty (Co-Construction by Ministry and Province), School of Food Science and Technology, Shihezi University, Shihezi 832000, China; 20222111016@stu.shzu.edu.cn (N.K.); zhexin_fan@shzu.edu.cn (Z.F.); shenjie@stu.shzu.edu.cn (J.S.); shenyuechenfei@stu.shzu.edu.cn (Y.S.); zhifengf@jiangnan.edu.cn (Z.F.); libaokun@shzu.edu.cn (B.L.); 2 Key Laboratory for Food Nutrition and Safety Control of Xinjiang Production and Construction Corps, School of Food Science and Technology, Shihezi 832000, China; 3 Engineering Research Center of Storage and Processing of Xinjiang Characteristic Fruits and Vegetables, Ministry of Education, School of Food Science and Technology, Shihezi University, Shihezi 832000, China; 4 Functional Food Center, Key Laboratory of Xinjiang Medicinal Plant Resources Utilization, Ministry of Education, Shihezi University, Shihezi 832000, China; 5 Alashankou Customs Technology Center, Alashankou 833418, China; 18997761304@163.com; 6 State Key Laboratory of Food Science and Resources, School of Food Science and Technology, Jiangnan University, Wuxi 214122, China; bo.yang@jiangnan.edu.cn; 7 International Joint Research Laboratory for Maternal-Infant Microbiota and Health, Jiangnan University, Wuxi 214122, China

**Keywords:** milk fat globule membrane protein, camel milk fat, ulcerative colitis, gut microbiota, amino acid metabolism

## Abstract

The protective effects of the milk fat globule membrane (MFGM) in alleviating inflammation have been reported. However, limited attention has been paid to the key fraction of milk fat globule membrane protein (MFGMP). This study investigated the protective effects of camel MFGMP against dextran sulfate sodium (DSS)-induced ulcerative colitis (UC) in mice. The results revealed that administering 50 mg/kg MFGMP significantly alleviated colonic inflammation, as evidenced by a marked decrease in IL-6, IL-1β, and TNF-α levels, along with pathological damage in DSS-induced mice with UC. MFGMP supplementation partially regulated gut microbiota dysbiosis in mice with UC by increasing α-diversity and the relative abundance of beneficial gut bacteria, such as *Lactobacillus*, while decreasing the abundance of *Akkermansia*. Additionally, MFGMP treatment exhibited significant regulatory effects on metabolites, particularly amino acid metabolism, in the feces. Specifically, this treatment restored L-valine to normal physiological levels and increased the concentrations of L-leucine, L-lysine, and L-tyrosine to nearly twice their baseline levels, whereas the concentration of L-tryptophan increased threefold. These upregulated amino acids were negatively correlated with pro-inflammatory cytokines and positively correlated with the anti-inflammatory cytokine IL-10, as indicated by Spearman’s correlation analysis. Furthermore, the significant reduction in the mRNA expression levels of WNT-1, β-catenin, and Cyclin D1 suggests that MFGMP exerts a positive effect on UC via the Wnt/β-catenin pathway. These findings indicate that MFGMP exerts a protective effect against UC by modulating intestinal microbiota and amino acid metabolism in mice, with potential implications for treating intestinal inflammatory diseases.

## 1. Introduction

Ulcerative colitis (UC) is a chronic inflammatory bowel disease (IBD) marked by symptoms such as weight loss, loose stools accompanied by hematochezia, diarrhea, and a dysregulated immune response [1]. Research indicates that the rising incidence of UC among children and adolescents points to the persistence of numerous environmental factors that contribute to increased susceptibility [2]. A family history of UC has been identified as the most significant risk factor for the disease [3]. Moreover, immune system dysfunction—characterized by T-cell abnormalities and an exaggerated immune response—has been linked to an elevated risk of developing UC [4]. These findings highlight the potential benefit of additional prophylactic measures for individuals at heightened risk, particularly those with a family history of UC or those chronically exposed to environmental and lifestyle factors. The medical community still lacks clarity regarding the pathological mechanisms of UC [5]. Although the exact mechanisms of UC are complex and remain unclear, it is mainly associated with intestinal disorders, including disrupted gut microbiota, gut inflammation, intestinal barrier dysfunction, and imbalanced immune responses. Promising strategies for the management of UC involve targeting the gut microbiota and its associated metabolites. There is widespread agreement that the gut microbiota plays a pivotal role in modulating UC. Recently, dietary and nutritional interventions, including proteins, probiotics, and polysaccharides, have been considered effective strategies to regulate UC. The relationship between UC and milk has gained increasing attention, with studies demonstrating that restricting milk production exacerbates UC disease.

Camel milk contains abundant nutrients, with higher lactoferrin and calcium levels, and is free of β-lactoglobulin (BLG) compared to bovine milk, making it a better mimic of breast milk in terms of nutrient composition [6]. Camel milk is generally used as a natural health supplement because of its superior antioxidant, immunomodulatory, anti-inflammatory, antidiabetic, and anti-apoptotic properties [7,8]. The milk fat globule membrane (MFGM) is a tri-layered protein–lipid structure that envelops fat globules. MFGM has gained increasing attention owing to its remarkable properties, including promotion of lipid uptake [9], restoration of intestinal barrier function [10], anti-inflammatory effects [11] and enhanced gut health [12]. Orally administering a combination of MFGM, fructo-oligosaccharides (FOSs), and galacto-oligosaccharides (GOSs) significantly improves UC in mice, primarily due to changes in the intestinal microbiota [13]. Studies have demonstrated that MFGM extracted from bovine milk can prevent and attenuate acute UC in mice. Moreover, several studies have suggested that MFGM modulates the immune response, strengthens intestinal barrier function, and promotes the diversity of the gut microbiota, thereby alleviating UC lesions [11].

Given that MFGM is a complex fraction containing 25–70% protein and a significant amount of lipids, it is difficult to determine which component is responsible for its biological functions. MFGMP, which includes epidermal growth factor 8 (EGF8), xanthine oxidoreductase (XOR), mucin 1 (MUC1), mucin 15, CD36, butyrophilin, and fatty acid-binding proteins, is highly regarded for its extensive biological functions despite being present in low amounts. Currently, studies on MFGMP have mainly focused on the variations in composition among different species and their protective effects on health. For example, XOR has been shown to contribute to mammary gland development and exert antimicrobial activity in the intestine, while proteins from the butyrophilin family are implicated in immunomodulation [14]. Moreover, MUC1, a member of the mucin family, plays a key role in protecting the mucosal barrier from pathogenic attack and is strongly associated with tumorigenesis, proliferation, and metastasis [15]. Recent studies have depicted that EGF8 from bovine milk MFGM can attenuate UC by increasing the production of butyric acid and improving intestinal microbiota in the cecal content, suggesting that EGF8 may be a key functional constituent in regulating gut health. Despite the high abundance of EGF8 in camel milk, it is widely accepted that the composition of MFGMP varies among species, as evidenced by the different isoforms of EGF8 found in bovine and camel milk. However, to date, no studies have explored the potential protective role of MFGMP in UC. Consequently, the role of MFGMP in camel milk warrants further investigation.

To understand the role of MFGMP in UC treatment, we administered MFGMP via gavage to mice with UC induced by dextran sulfate sodium salt (DSS). The objectives of this study were to (1) investigate the effects of MFGMP on UC, (2) analyze changes in gut microbiota and metabolites, (3) determine the amount of short-chain fatty acids (SCFAs) in the cecum, and (4) determine the correlation between differential metabolites, gut microbiota, and inflammatory cytokines. By elucidating the underlying mechanisms, these findings lay the foundation for the future application of MFGMP as a functional food in the relief and management of ulcerative colitis.

## 2. Materials and Methods

### 2.1. Preparation of MFGM Proteins

Six mature camel milk samples were randomly collected from a local Bactrian camel farm in Shihezi City, Xinjiang Uygur Autonomous Region, China. All samples were pooled, placed in iceboxes, and immediately transported to the laboratory, where fresh raw camel milk was used for MFGMP preparation. MFGMP was prepared according to the method outlined by Dou et al. [16], with minor modifications. Raw milk samples were centrifuged at 8000 revolutions per minute (rpm) for 20 min at 4 °C. The upper layer, consisting of the cream, was carefully removed with a spatula and rinsed three times with phosphate-buffered saline (PBS) at pH 7.4. The milk fat globules were suspended in ultrapure water, incubated at 37 °C for 10 min to remove any residual PBS, and centrifuged at 10,000 rpm for 15 min, and the upper layer of milk fat was collected and crystallized at 4 °C overnight. Ultrapure water was added to the MFGM fraction, and the MFGM was homogenized by sonication in an ultrasonic cell disruption machine (sonication 3 s, interval 9 s, total 20 min). Homogenized MFGM was incubated in a shaker at 45 °C for 30 min to promote cream melting, followed by centrifugation at 12,000 rpm for 15 min at 4 °C. The resulting residue was identified as MFGMP.

### 2.2. Experimental Design and Animal Model

**Figure 1:**
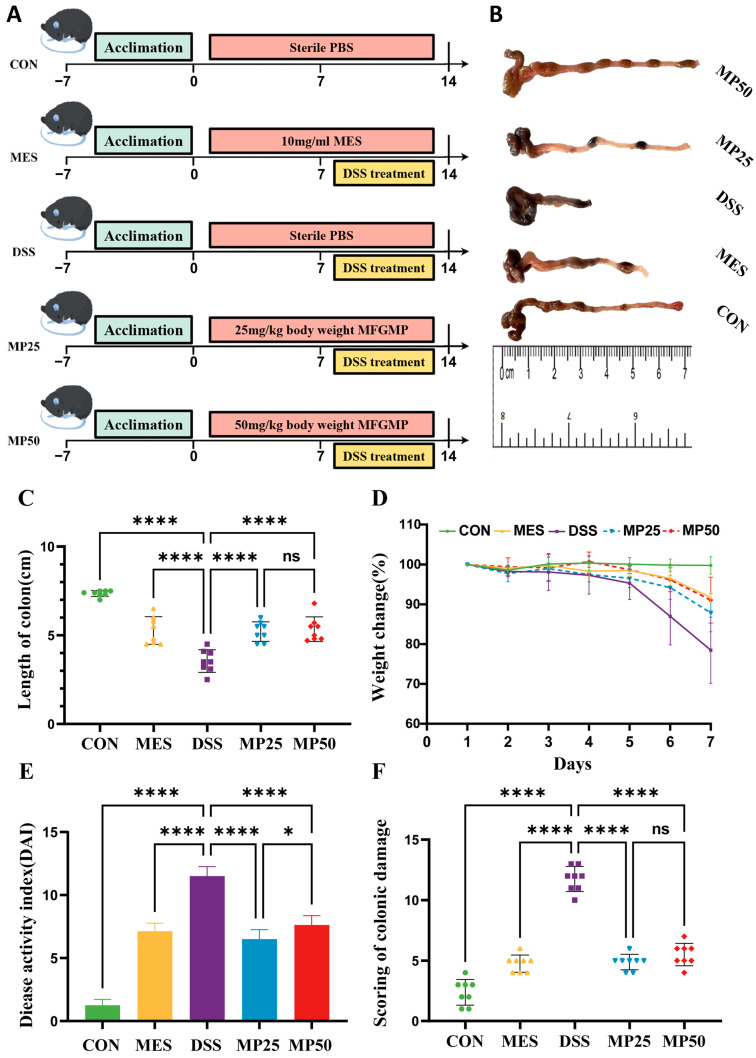
MFGMP alleviated DSS-induced UC in mice. (**A**) The diagram showcases this study’s experimental design. For two weeks, mice received either oral PBS or MFGMP treatment prior to being provided with drinking water that contained 3% DSS. (**B**) Macroscopic images of the colon. (**C**) Colon length in each group (n = 6–8 per group). (**D**) Change in daily body weight during DSS treatment. (**E**) Kinetics of daily disease activity index (DAI) scores throughout DSS treatment. (**F**) Histological scores of the colon samples. Data are presented as mean ± standard deviation (SD) (n = 68 per group). Statistical significance was determined using a one-way analysis of variance (ANOVA), followed by Tukey’s test. ns, non-significant, * *p* ≤ 0.05 and **** *p* ≤ 0.001 relative to the CON group.

The animal protocol (A2023-087) was approved by the Ethics Committee of Laboratory Animals at the University of Shihezi, Xinjiang, China. Forty specific pathogen-free male C57BL/6 mice (six weeks old) were obtained from SCBS Biotechnology Co., Ltd. (Henan, China) and housed in cages at the Experimental Animal Center of Shihezi University, where the environment maintained 12:12 h light–dark cycles at 20–25 °C and 60 ± 5% humidity. After a week of acclimatization, the mice were randomly assigned to five groups (n = 8): control group (CON), DSS-induced colitis group (DSS), mesalazine treatment group (MES), low MGFMP treatment group (MP25, 25 mg/kg), and high MGFMP treatment group (MP50, 50 mg/kg). UC was induced using 3.0% (*w*/*v*) DSS in drinking water for 7 days according to previously described protocols [17]. The experimental design is illustrated in Figure 1A. After a 12 h fast, all mice were anesthetized with ether and euthanized by cervical dislocation at the end of the experiment.

### 2.3. Colitis Scores and Histological Analysis

The DAI was employed daily to evaluate the colitis scores in the mice, which encompassed factors such as weight loss, stool consistency, and rectal bleeding. These parameters were monitored as previously described. Colon length was measured after the mice were sacrificed. Samples from the distal colon were preserved in 4% paraformaldehyde (Xavier Biotechnology Co., Ltd., Wuhan, China), dehydrated using ethanol, and embedded in paraffin to create sections that were 5 μm thick. The sections underwent staining with hematoxylin and eosin (H&E) along with Alcian blue dye to facilitate histopathological analysis [18]. The tissue damage in each group of colon tissue sections was scored using Dieleman’s scoring system, which evaluated four aspects: lesion extent, crypt destruction, lesion depth, and severity of inflammation [19].

### 2.4. Analysis of Inflammatory Cytokines

The serum levels of IL-6, IL-1β, TNF-α, and IL-10 were quantified using ELISA kits (Enzyme-linked Biotechnology Co., Ltd., Shanghai, China).

### 2.5. Analysis of Relative mRNA Expression in Colonic Tissues

Total RNA was extracted from colonic tissues using TRIzol (Invitrogen, Carlsbad, CA, USA) reagent following the manufacturer’s protocol. Real-time PCR was conducted with SYBR Green PCR Master Mix (Thermo Fisher Scientific, Waltham, MA, USA) according to the manufacturer’s instructions. β-Actin was used as the reference gene, and relative gene expression was calculated using the 2−ΔΔCt method. The primer sequences employed in this study are provided in Table S1.

### 2.6. Determination of Short-Chain Fatty Acids (SCFAs)

SCFAs were extracted using a method similar to that previously reported, with slight modifications [20]. Briefly, 30 mg of cecal content was transferred into a 2 mL tube, followed by the addition of 1.5 mL of ultrapure water. The mixture was vortexed for 30 s, then centrifuged at 5000 rpm for 4 min (room temperature). The supernatant was transferred to a new 2.0 mL tube and mixed with 100 μL of 25% metaphosphoric acid by vortexing for 30 s. After centrifugation at 15,000 rpm (room temperature), the supernatant was collected in an injection vial for gas-phase analysis. SCFAs in the cecal content were quantified by gas chromatography using a 30 m × 0.25 mm × 0.50 μm DB-WAX column (Agilent Technologies, PaloAlto, CA, USA).

### 2.7. Immunohistochemistry

Colonic tissue slides (4 μm) were deparaffinized and hydrated. Antigen retrieval from formalin-fixed tissues was performed by microwave heating (20 min) with 0.01 M citric acid. Tissue blocking was carried out using 5% bovine serum albumin for 1 h at 37 °C. Immunohistochemistry was performed using primary antibodies against WNT-1 (27935-1-AP), β-catenin (17565-1-AP), and Cyclin D1 (26969-1-AP) purchased from LifeScience Biological Experimental Equipment Co., Ltd (Wuxi, China). HRP-conjugated goat anti-rabbit IgG secondary antibody (G1213-100UL, Xavier Biotechnology Co., Ltd.) was used for detection. Antibodies were detected using a 3,3′-diaminobenzidine (DAB) staining kit (G1212-200T, Xavier Biotechnology Co., Ltd.). Hematoxylin served as a counterstain, and the slides were covered with neutral balsam and a coverslip.

### 2.8. Microbial Community Analysis

Total DNA from feces was extracted using the FastDNA^®^ Spin kit (MP Biomedicals, Santa Ana, CA, USA) for soil following the manufacturer’s instructions to ensure high-quality DNA and accuracy of analysis. Universal primers 338F (5′-ACTCCTACGGAGGCAGCAG-3′) and 806R (5′-GGACTACHVGGGTWTCTAAT-3′) were used to amplify the V3–V4 region of the gene [21]. The samples were identified using barcodes and primers, and the sequences were dereplicated and discarded. The data were sequenced using the Miseq PE300 platform (Illumina, San Diego, CA, USA) and then searched for libraries. Raw FASTQ files were demultiplexed, quality-filtered using Trimmomatic (version 0.3.9), and merged using fast length adjustment of short reads to improve genome assembly. Bioinformatic analysis was performed using Tutu Cloud Platform (http://cloudtutu.com.cn/, accessed on 13 August 2024) and MICROKOM BioScience Cloud (https://www.bioincloud.tech, accessed on 13 August 2024) OECloud tools.

### 2.9. Metabolomics Analysis

Fecal samples from mice were combined with an extraction solution consisting of methanol and water in a 4:1 ratio (*v*:*v*) for the purpose of metabolite extraction. This mixture underwent low-temperature ultrasonic extraction for 30 min at 5 °C and 40 kHz, followed by centrifugation at 4 °C and 13,000× *g*. The resulting supernatant was transferred into injection vials equipped with internal cannulae for subsequent online analysis using LC-MS/MS (Thermo Fisher Scientific, USA). Analysis using LC-MS was conducted with a UHPLC-Q Exactive HF-X (Thermo Fisher Scientific, USA) system. This was succeeded by peak alignment, correction of retention times, and extraction of peak areas using Progenesis QI (Milford Waters, Milford, MA, USA) [22]. Data were matched to metabolic databases and uploaded to the Meggie BioCloud (https://cloud.majorbio.com, accessed on 17 August 2024) platform for analysis.

### 2.10. Statistical Analysis

Data are presented as mean ± SD. The analyses of all data were conducted using GraphPad Prism software (version 9.0.0). For statistical evaluation, a one-way ANOVA followed by Tukey’s multiple comparison test was employed. A statistical significance threshold was established at * *p* < 0.05, ** *p* < 0.01, and *** *p* < 0.001. Values with ns indicate non-significance.

## 3. Results

### 3.1. MFGMP Mitigates the Symptoms of DSS-Induced UC in Mice

To assess the therapeutic potential of MP25 and MP50 for UC, key indicators of UC were characterized. The symptoms of DSS-induced UC were similar to those of human colitis. Mice in the DSS group exhibited a notable reduction in body weight and colon length. The symptoms of UC, including reduced colon length and weight loss, significantly improved in the MP25 and MP50 groups, with no notable differences between them (Figure 1B–D). However, MP25 significantly improved the DAI index compared to MP50 (Figure 1E).

### 3.2. MFGMP Attenuates Colon Pathology Symptoms of DSS-Induced UC in Mice

**Figure 2:**
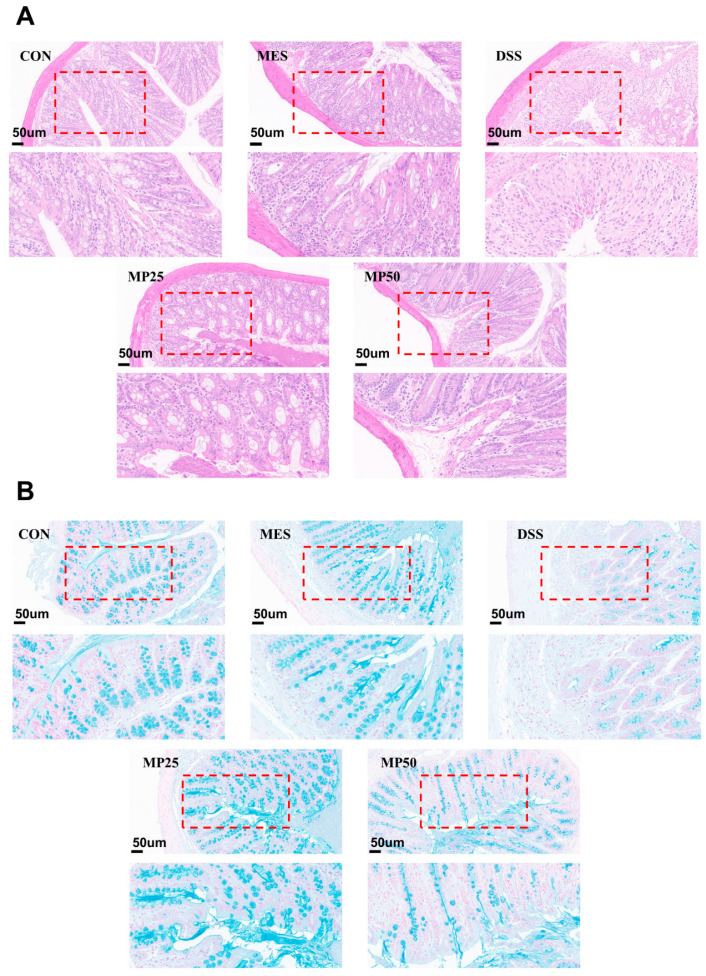
Colonic tissue staining sections. (**A**) H&E-stained colon sections (n = 6 per group) with scale bars representing 50 µm. (**B**) Alcian blue-stained colon sections (n = 6 per group), with scale bars representing 50 µm.

Damage to colonic tissue can be assessed by H&E staining. The colonic mucosa of the mice in the DSS group was nearly destroyed, with the crypts and goblet cells absent and marked inflammatory cell infiltration compared to the CON group. The colonic tissues of the mice in the MP25 and MP50 groups exhibited improvement, with tissues close to normal. Crypts in the MP25 group were not damaged and contained abundant goblet cells, resulting in better recovery than those in the MP50 group. Consequently, MFGMP alleviated DSS-induced colonic injury and protected the intestinal mucosa, especially in the MP25 group (Figure 2A). The colon histopathology score was based on the evaluation of key pathological features, including inflammatory cell infiltration, crypt disruption, and epithelial damage. Colon histopathology scores revealed significantly higher scores in the DSS group compared to the CON group (Figure 1F). The MES, MP25, and MP50 groups exhibited significantly lower scores than the DSS group, with no significant difference between the MP25 and MP50 groups.

### 3.3. MFGMP Relieves the Amount and Distribution of Mucin in DSS-Induced UC in Mice

The mucus layer, the first barrier of the colon against harmful substances and microorganisms, consists of an inner layer of polymerized MUC2 and an outer layer of loosely packed MUC2. MUC2 is secreted by cup cells and forms a mesh-like structure that prevents microbial invasion and maintains intestinal homeostasis [23]. The distribution of mucin MUC2 was analyzed by Alcian blue staining, and the mucus layer was severely disrupted, with mucin significantly reduced in the DSS group. However, MES, MP25, and MP50 groups demonstrated improved mucin distribution and less damage to the intestinal mucus layer in the UC mice. Interventions with MGFMP significantly increased the mucin amount and restored mucus secretion. The mucin content in the MP25 group was significantly higher than in the MP50 group (Figure 2B). Tissue section staining demonstrated that MP25 was more effective than MP50 in alleviating localized inflammation and restoring intestinal injury.

### 3.4. MFGMP Modulates Systemic and Intestinal Local Inflammation in DSS-Induced UC in Mice

**Figure 3:**
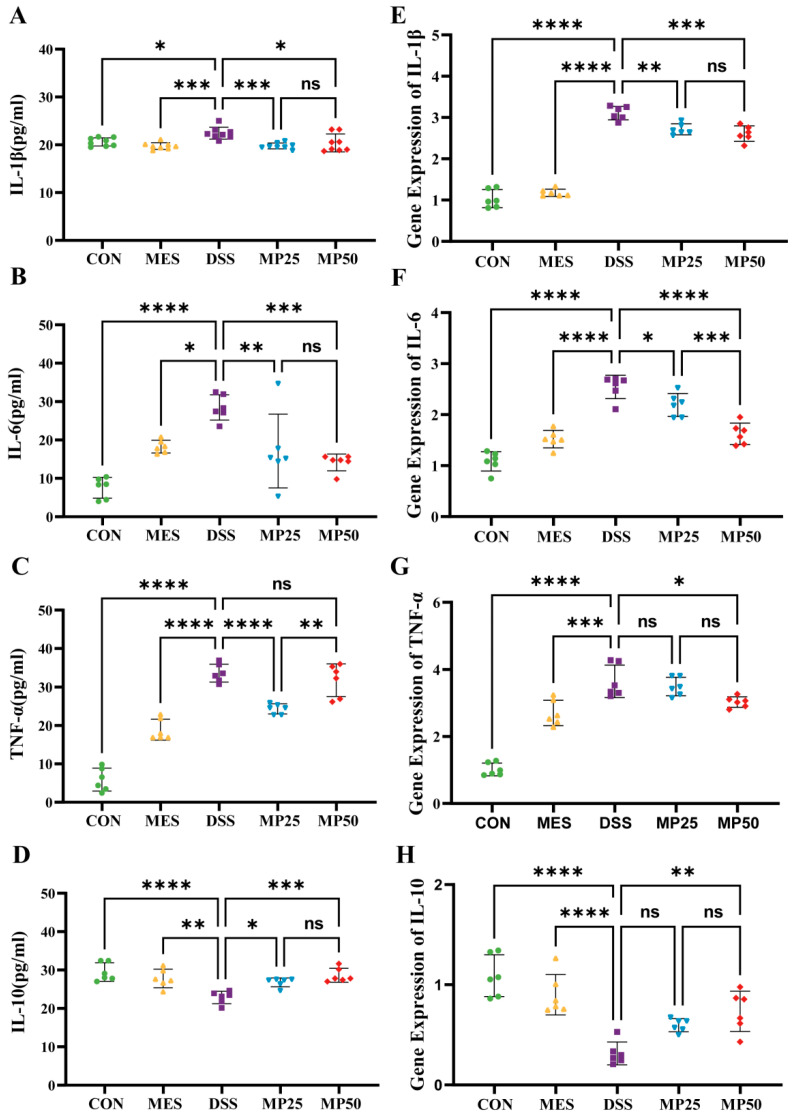
The concentrations of IL-6, IL-1β, TNF-α, and IL-10 and their mRNA expression levels were determined using ELISA kits and real-time PCR. Concentrations of four representative cytokines: IL-1β (**A**), IL-6 (**B**), TNF-α (**C**), and IL-10 (**D**) in the serum. The mRNA levels of four representative cytokines, IL-1β (**E**), IL-6 (**F**), TNF-α (**G**), and IL-10 (**H**), in colon tissues from the indicated groups were estimated by real-time quantitative PCR. Data are presented as mean ± SD (n = 6–8 per group). Statistical significance was determined using one-way ANOVA, followed by Tukey’s test. ns, non-significant, * *p* ≤ 0.05, ** *p* ≤ 0.01, *** *p* ≤ 0.005, and **** *p* ≤ 0.001 relative to CON group.

Since UC patients are often associated with immune dysregulation, the effect of MGFMP on both systemic and localized intestinal inflammation was further investigated. To evaluate the effect of MGFMP on systemic inflammation, the serum levels of IL-6, IL-1β, IL-10, and TNF-α were quantified using ELISA. The results showed that the MP25 group had significantly lower levels of pro-inflammatory cytokines, including IL-6, IL-1β, and TNF-α, compared to the DSS group. The MP50 group, meanwhile, exhibited significantly decreased levels of IL-6 and IL-1β (Figure 3A–C). Both MFGMP treatments resulted in a significant increase in the levels of the anti-inflammatory cytokine IL-10 (Figure 3D). Notably, the pro-inflammatory cytokine TNF-α levels in the MP25 group were significantly lower than those in the MP50 group.

RT-PCR was conducted to further examine the effect of MGFMP on localized intestinal inflammation. The mRNA expression levels of IL-6, IL-1β, and TNF-α in the DSS-induced UC group were significantly higher than those in the CON group. The MP25 and MP50 groups demonstrated significantly suppressed mRNA expression levels of IL-6 and IL-1β, which was consistent with the changes in cytokines observed in the serum. However, the mitigating effects on TNF-α and IL-10 were associated with the concentration of MFGMP, with significant effects observed only in the MP50 group (Figure 3E–H). The MP25 and MP50 groups demonstrated alleviated systemic inflammation, whereas only the MP50 group demonstrated a more pronounced effect on intestinal localization. Therefore, we explored the mechanism underlying the mitigation of DSS-induced UC through gavage administration of 50 mg/kg MFGMP.

### 3.5. Effect of MFGMP on the Intestinal Microbiota of DSS-Induced UC in Mice

**Figure 4:**
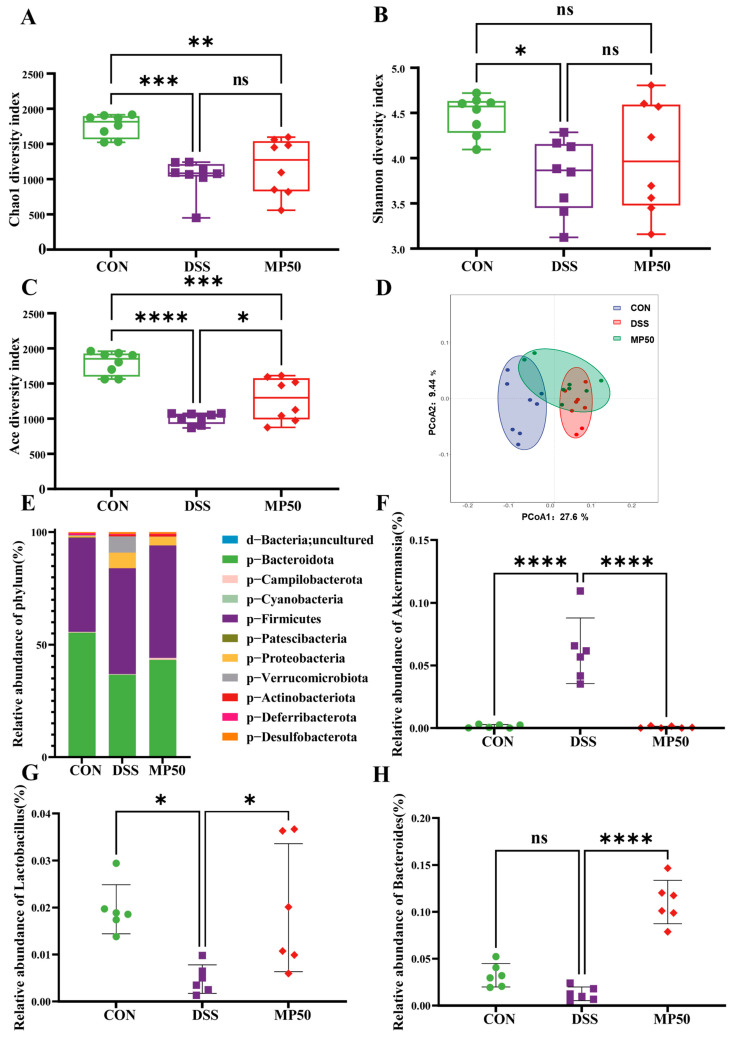
Effects of MFGMP on the composition of the gut microbiota. (**A**) Alpha diversity Chao1 index (n = 8). (**B**) Alpha diversity Shannon index (n = 8). (**C**) Alpha diversity ACE index (n = 8). (**D**) Principal coordinate analysis of weighted UniFrac distances. (**E**) Community structure analysis for each group at the phylum level. (**F**) Relative abundance of *Akkermansia* species (**G**) Relative abundance of *Lactobacillus* species. (**H**) Relative abundance of *Bacteroides* species. Statistical significance was determined using one-way ANOVA, followed by Tukey’s test. ns, non-significant, * *p* ≤ 0.05, ** *p* ≤ 0.01, *** *p* ≤ 0.005, and **** *p* ≤ 0.001 relative to CON group.

UC is usually associated with changes in the composition and diversity of the gut microbiota [24]. Therefore, the effect of MFGMP on the intestinal flora of UC mice was investigated. DSS treatment decreased the α-diversity of the gut microbiota in mice, with Chao1, ACE, and Shannon indices significantly lower than those of the CON group. Orally administering MFGMP significantly restored the ACE index (Figure 4A–C). The β-diversity of intestinal bacteria depicted that mice in the DSS group were significantly different from mice in the CON group. After MFGMP treatment, intestinal bacteria tended to approach those in the CON group (Figure 4D).

At the phylum level, the relative abundance of Bacteroidota, Firmicutes, and Verrucomicrobiota was significantly different from that in CON group, with significant reversal of the change by MFGMP intervention (Figure 4E). At the genus level, MFGMP intervention significantly decreased the relative abundance of *Akkermansia* (Figure 4F) and *Clostridia_UCG-014* compared to the DSS group. In contrast, the relative abundance of *Lactobacillus* (Figure 4G), *Bacteroides* (Figure 4H), and *Alloprevotella* increased significantly.

**Figure 5:**
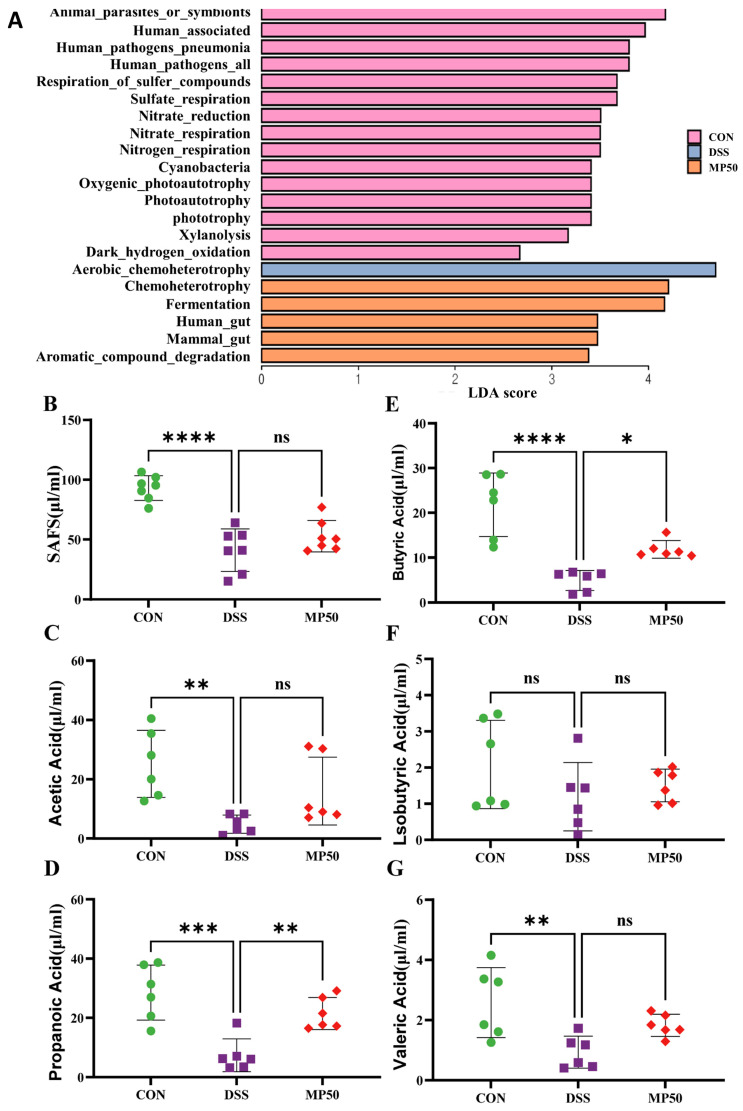
Determination of SCFAs in the cecum using gas chromatography–mass spectrometry. (**A**) Microbial gene functions predicted using FAPROTAX based on LDA scores. Total SCFAs (**B**), acetic acid (**C**), propanoic acid (**D**), butyric acid (**E**), isobutyric acid (**F**), and valeric acid (**G**) contents in mouse feces (n = 6). ns, non-significant, * *p* ≤ 0.05, ** *p* ≤ 0.01, *** *p* ≤ 0.005, and **** *p* ≤ 0.001 relative to CON group.

The gene functions of known microorganisms were predicted using FAPROTAX (version 1.2.7) software, revealing that the gut microbiota in the DSS group was enriched in aerobic chemoheterotrophy. MFGMP interfered with the microbial function of chemoheterotrophy, as well as the functions of fermentation, the human gut, the mammalian gut, and automatic compound degradation (Figure 5A).

### 3.6. Effect of MFGMP on SCFAs of DSS-Induced UC in Mice

SCFAs are metabolic products of gut microbiota and play a crucial role in gut health and immune regulation. UC induced a decrease in SCFAs in the cecal content (Figure 5B). However, butyric acid and propionic acid significantly increased after MFGMP application compared to the DSS group, although the effect on total SCFAs was non-significant (Figure 5C,E). This suggested that the relief of inflammation by MFGMP may be related to butyric and propionic acids.

### 3.7. Effect of MFGMP on Intestinal Metabolites of DSS-Induced UC in Mice

**Figure 6:**
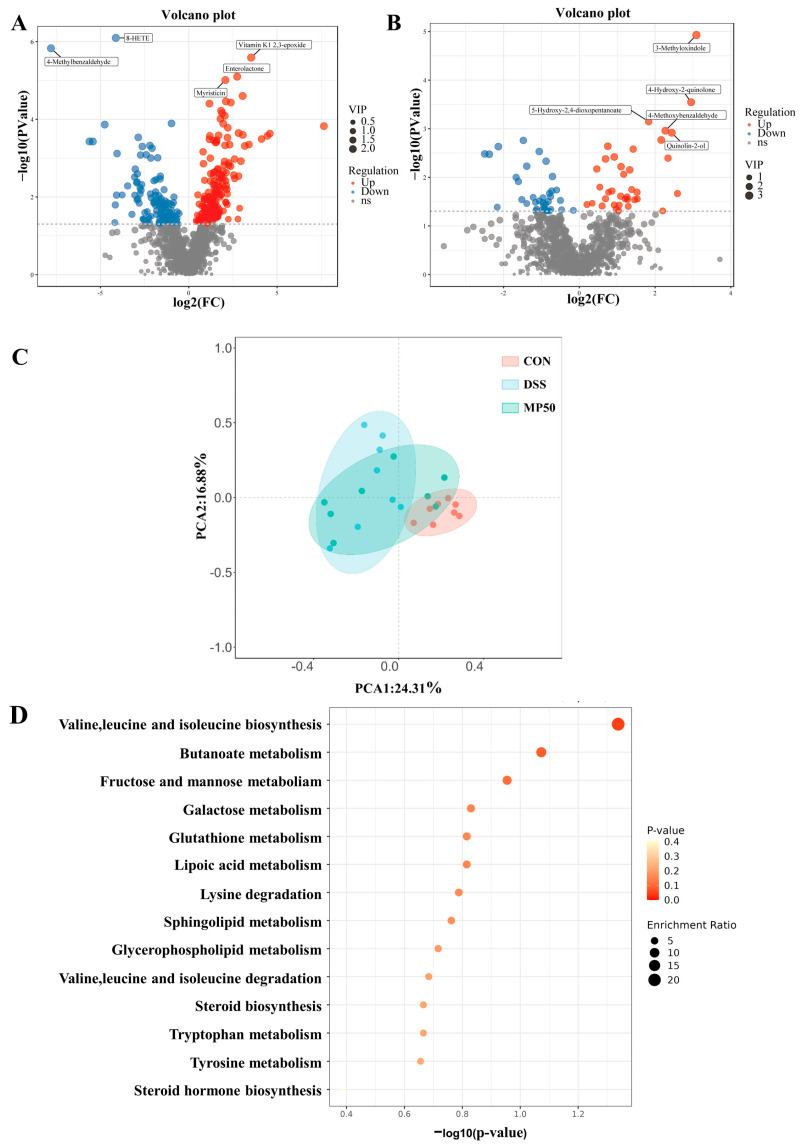
MFGMP altered metabolic profiles in DSS-induced UC. (**A**) Volcano map reflecting these variables between the CON and DSS groups. (**B**) Volcano map reflecting these variables between the DSS and MP50 groups. (**C**) Bubble diagram of KEGG enrichment analysis of 24 secondary differential metabolites. (**D**) PCA scores of the differential metabolites in the CON, DSS, and MP50 groups.

Gut microbial metabolites are important molecular mediators between the host and gut microbes and have important and diverse effects on host physiology. A total of 12,991 metabolites in the cecal contents were analyzed using an untargeted metabolomics approach, and 181 major metabolites were identified as significantly different based on VIP > 1 (*p* < 0.05). Twenty-four key differential metabolites were identified by screening different metabolites in the DSS and CON groups, and metabolites that were significantly regulated by MFGMP gavage were further screened (Table S2). Principal component analysis (PCA) of the metabolites from the three groups of mice demonstrated that the model was highly interpretable and effective in differentiating between them (Figure 6A,B). PCA analyses of the CON, DSS, and MP50 groups depicted significant differences, with the MP50 group shifting from the DSS group to the CON group, which was located in between. This observation suggests that MFGMP may reduce the effects of DSS modeling (Figure 6C).

MetaboAnalyst (www.metaboanalyst.ca, accessed on 15 August 2024) was employed to perform KEGG pathway enrichment analysis on the list of differential metabolites [25]. The results depicted that MFGMP regulates 14 major metabolic pathways in a mouse model of UC (Figure 6D), particularly related to the metabolic pathways of amino acids, which encompass the biosynthesis of valine, leucine, and isoleucine; the degradation of lysine; the breakdown of valine, leucine, and isoleucine; as well as the metabolism of tryptophan and tyrosine.

### 3.8. Impact of MFGMP on Amino Acid Metabolism in DSS-Induced UC in Mice

**Figure 7:**
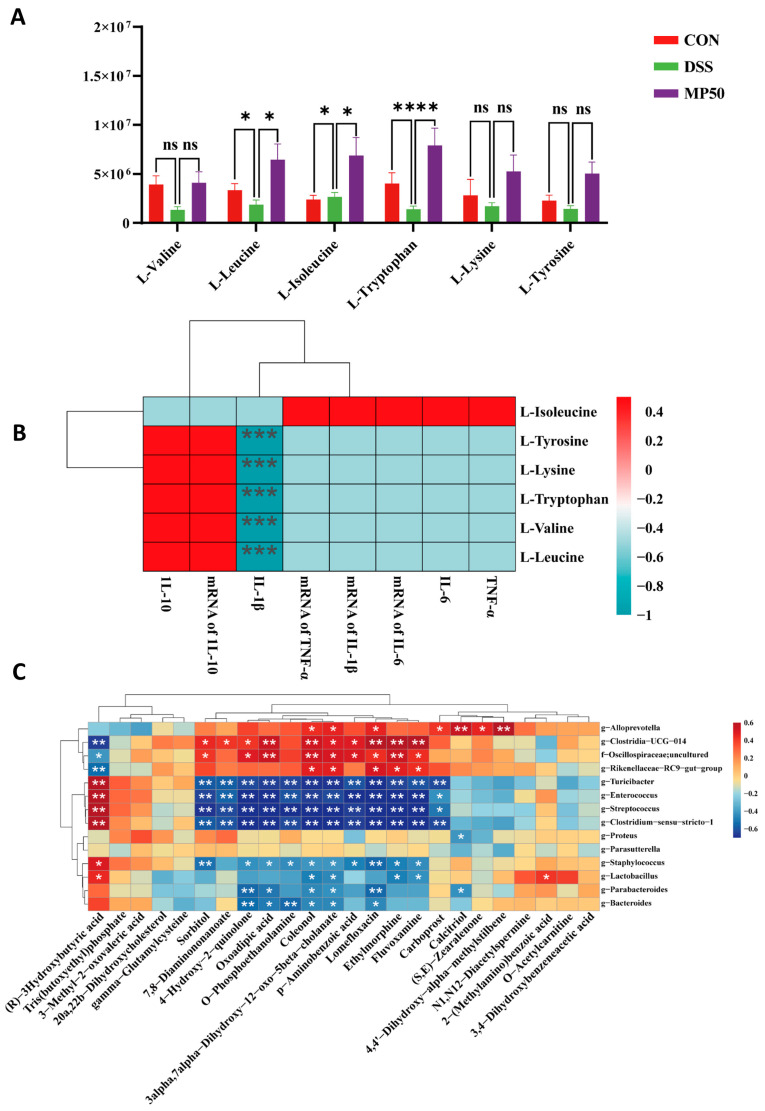
Effects of MFGMP on amino acid metabolism in mice with DSS-induced UC. (**A**) Specific amino acid content in mouse feces. (**B**) Heatmap of the correlation between the amino acid content in feces and cytokines. (**C**) Heatmap of the correlation between metabolites and microbiota composition in mice with DSS-induced UC. ns, non-significant, * *p* < 0.05, ** *p* < 0.01, and *** *p* < 0.005.

Some studies have found that the metabolism of specific amino acids may influence intestinal immune and inflammatory responses and that UC symptoms can be reduced by adjusting amino acid metabolism [26]. The levels of specific amino acids involved in the amino acid pathway were analyzed. The amino acid levels of L-valine, L-leucine, L-tryptophan, L-lysine, and L-tyrosine were lower in the DSS group compared to the CON group. After administering MFGMP through gavage, the level of L-valine returned to normal, while the concentrations of L-leucine, L-lysine, and L-tyrosine increased to nearly twice their normal levels, and the concentration of L-tryptophan increased to three times its original level (Figure 7A).

A correlation analysis was performed to investigate the relationship between amino acid content and inflammatory responses. L-valine, L-leucine, L-tryptophan, L-lysine, and L-tyrosine levels were negatively correlated with the mRNA expression levels of three pro-inflammatory cytokines (IL-6, IL-1β, and TNF-α). L-isoleucine was positively correlated with the mRNA expression levels of the four pro-inflammatory cytokines. L-valine, L-leucine, L-tryptophan, L-lysine, and L-tyrosine levels were positively correlated with the anti-inflammatory cytokine IL-10 and its mRNA expression levels. L-isoleucine was negatively correlated with IL-10 and its mRNA expression levels (Figure 7B). MFGMP inhibits inflammation primarily by regulating amino acid metabolic pathways.

### 3.9. Correlation Between Metabolites and Microbiota Composition

The top 20 differential microbial genera were filtered using fold change (FC) values (FC > 2 or FC < 0.5), and correlations with the previously identified 24 differential metabolites were assessed using Spearman’s correlation analysis (Figure 7C). (R)-3-Hydroxybutyric acid, which is associated with butyric acid metabolism, was significantly positively correlated with *Lactobacillus* and *Clostridium_sensu_stricto*. There was a highly significant positive correlation between *Clostridia_UCG-014* and oxoadipic acid, fluvoxamine, ethylmorphine, and lomefloxacin, particularly oxoadipic acid, which is a product of tryptophan metabolism (*p* < 0.01). 4,4′-Dihydroxy-alpha-methylstilbene, coleonol, and Alloprevotella demonstrated a highly significant positive correlation (*p* < 0.01). MFGMP-derived metabolites, such as 3,4-dihydroxyphenylacetic acid, 3-methyl-2-oxovaleric acid, and oxoadipic acid, play important roles in tyrosine metabolism as well as valine, leucine, and isoleucine biosynthesis and tryptophan metabolism.

### 3.10. Effect of MFGMP on Wnt/β-Catenin Signaling Pathway in Mice Model

**Figure 8:**
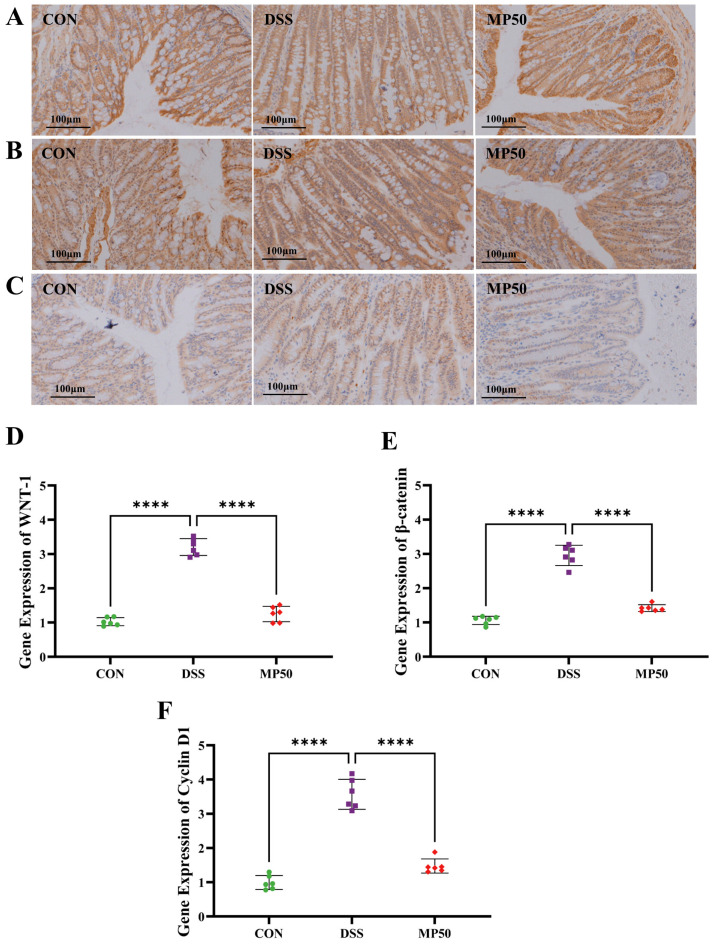
MFGMP downregulates the WNT-1-β-catenin-Cyclin D1 signaling pathway to alleviate inflammatory responses. Immunohistochemical staining for WNT-1 (**A**), β-catenin (**B**), and Cyclin D1 (**C**). Scale bars, 100 μm. The mRNA levels of three representative genes, WNT-1 (**D**), β-catenin (**E**), and Cyclin D1 (**F**), in colon tissues from the indicated groups were measured by real-time quantitative PCR. Statistical significance was determined using one-way ANOVA, followed by Tukey’s test. **** *p* ≤ 0.001 relative to the DSS group. The data are presented as mean ± SD (n = 6 per group).

WNT-1 regulates cell proliferation and differentiation by activating β-catenin. In DSS-induced UC, β-catenin accumulates in the cytoplasm of cells and migrates to the nucleus due to inflammation, activating the expression of various genes such as Cyclin D1, which further promotes cell proliferation and inflammation [27,28]. DSS treatment significantly increased the expression of WNT-1, β-catenin, and Cyclin D1, as indicated by the immunohistochemical staining results (Figure 8A–C). RT-qPCR analysis revealed that the mRNA levels of WNT-1, β-catenin, and Cyclin D1 were significantly lower after MFGMP gavage compared to the DSS group (Figure 8D–F). MFGMP may alleviate DSS-induced UC through the Wnt/β-catenin signaling pathway.

## 4. Discussion

MFGM is commonly removed when milk fat is separated through centrifugation in the process of creating infant formula derived from bovine milk. However, the protective effects of MFGM in reducing inflammation, enhancing immunity, and improving cognitive function have been well documented. MFGM proteins have emerged as promising nutritional components that offer significant health benefits to mammals across all life stages [29]. Recently, substantial progress has been made in the clinical applications of MFGM peptides. Studies have demonstrated that MFGMP can regulate lipid metabolism, mimicking the structural advantages of breast milk in infant formulas and enhancing digestive efficiency [30]. Additionally, clinical trials have demonstrated that MFGMP can lower the incidence of hemorrhagic diarrhea and support intestinal maturation in infants [31]. Given these results, it can be inferred that MFGMP holds considerable promise for expanded use in dairy processing.

The mitigating effect of MGFMP on UC is related to its concentration. A 50 mg/kg dose was more effective in mitigating UC in mice, particularly in preventing weight loss and alleviating localized intestinal inflammation. However, it cannot be overlooked that 25 mg/kg MFGMP was more effective in lowering the DAI index and serum TNF-α levels. The DAI is an extensive evaluation system that takes into account alterations in weight, stool consistency, and instances of bleeding. Both 25 and 50 mg/kg MFGMP significantly decreased the DAI. However, loose stools in the MP50 group resulted in higher DAI. Protein degradation and absorption are disrupted in the intestinal tract, which is inflamed or infected by pathogens because of the inhibited secretion of digestive enzymes [32]. Concurrently, pro-inflammatory cytokines interfere with digestion, enhance smooth muscle contraction of the intestinal wall, accelerate the passage of contents, reduce water absorption, increase gas production, and aggravate diarrhea [33]. Therefore, high protein concentrations may impose an additional burden on the inflamed bowel, eventually leading to loose stools [34]. The elevated TNF-α levels in the MP50 group may have been due to the accumulation of leucine in feces in this study. Research conducted in the past has indicated that leucine stimulates the mammalian target of the rapamycin (mTOR) signaling pathway, which in turn enhances the synthesis of TNF-α [35,36].

Gut microbiota dysbiosis is widely considered a key factor contributing to UC. Many investigations have revealed that the makeup and by-products of the gut microbiome vary considerably between individuals suffering from intestinal disorders and those who are healthy [37,38]. MFGM, present in infant formulas, influences the gut microbiome in children and provides a protective effect against inflammation in the intestines. In normal mice, the dominant phyla include Firmicutes, Bacteroidetes, Desulfobacterota, Deferribacterota, and Proteobacteria [39]. Following DSS administration in mice, the relative abundances of Firmicutes, Verrucomicrobia, Actinobacteria, Desulfovibrio, and Proteobacteria were significantly elevated. Conversely, there was a simultaneous decline in the relative abundance of Deferribacterota and Bacteroidetes [40]. The research indicated that the administration of MGFMP at a dosage of 50 mg/kg led to a notable rise in the prevalence of Bacteroidetes in comparison to the DSS group. Within the phylum Bacteroidetes, species such as *B. fragilis*, *B. ovatus*, and *B. thetaiotaomicron* are capable of producing metabolites such as kynurenine, indole-3-acetic acid, and indole-3-lactic acid [41]. These substances are the critical products of tryptophan metabolism. The intricate interplay between gut microbes and their by-products profoundly influences immune homeostasis, substantially affecting immune development and responses. This pivotal role in modulating UC pathogenesis underscores the critical importance of comprehensively understanding the dynamic relationships within the disease process [42].

The Firmicutes-to-Bacteroidota (F/B) ratio is a well-established factor in maintaining normal intestinal homeostasis. Following DSS administration, an increased F/B ratio was observed in the mice, indicative of dysbiosis. In contrast, the administration of MFGMP reduced the F/B ratio in mice suffering from DSS-induced ulcerative colitis, thus emphasizing the regulatory role of MFGMP in maintaining intestinal balance. Enrichment of the phylum Proteobacteria, which includes many potentially harmful bacteria, has been observed in patients with UC. This study demonstrated that MFGMP supplementation counteracted this trend, exhibiting a tendency to restore the microbial composition to that of the CON group.

Verrucomicrobiota, especially *Akkermansia*, plays a crucial role in regulating intestinal inflammation. The results of this study indicated a significant increase in *Akkermansia* abundance in the DSS group compared to the CON group. Administering 50 mg/kg MFGMP restored the relative abundance of *Akkermansia*, in line with previous studies [43]. Phospholipids, an important component of MFGM, promote an increase in *Akkermansia* in the intestine, suggesting that individual MFGM components may influence *Akkermansia* differently [10]. Additionally, milk fat globules from different animal sources exert varying effects on *Akkermansia* [44]. Further studies are needed to demonstrate the intricate interactions between *Akkermansia* and the MFGM. Some studies have reported a reduction in *Akkermansia* in colitic mice [45]. These findings suggest that the role of *Akkermansia* in IBD pathogenesis is complex and multifaceted. Several studies have demonstrated that *Akkermansia* exerts a pro-inflammatory effect under specific conditions, particularly when harmful bacteria damage the intestinal mucosal barrier, thereby enabling pathogens to invade the intestinal wall. This phenomenon triggers an intense immune response [46]. Notably, the species and number of pathogenic microorganisms can influence the delicate role of *Akkermansia* in IBD [47]. Several studies have demonstrated the anti-inflammatory properties of *Akkermansia*. Specifically, *Akkermansia* strengthens the intestinal barrier to prevent inflammation, indirectly inhibits chronic low-grade inflammation by regulating metabolism, activates Toll-like receptor 2 (TLR2) on the surface of intestinal epithelial cells to increase the release of anti-inflammatory factors, and restores normal tryptophan metabolism in patients with UC [48,49,50]. Consequently, the role of *Akkermansia* in IBD pathogenesis is influenced by various factors, including host genetics, gut microbiota composition, environmental conditions, and microbial interactions [51,52].

Previous studies have identified a positive correlation between *Lactobacillus* and tryptophan metabolism in patients with UC. In the MP50 group, upregulation of *Lactobacillus* and *Bacteroides* was observed compared to the DSS group, further supporting their role in regulating tryptophan metabolism in DSS-induced colitis.

SCFAs produced by intestinal microorganisms play a crucial role in maintaining the morphology and function of intestinal epithelial cells and serve as key regulators of UC [53]. This study demonstrated that the SCFA content in the appendix significantly decreased, which was akin to the decrease in SCFA levels reported in patients with UC [54]. Butyric acid serves as a vital energy source for colon cells, supports the transformation of macrophages to the M2 phenotype, aids in the healing of injured colonic epithelial cells, and is important for sustaining colon health. Following MFGMP administration, butyric and propionic acid levels were markedly elevated. This aligns with an earlier study conducted by Gong, et al. [55], which found that the supplementation of MFGM led to an elevation in the fecal concentrations of propionic acid, butyric acid, isobutyric acid, valeric acid, and isovaleric acid in a mouse model with BLG-induced allergies. It has been found that butyric acid has significant anti-inflammatory properties and can reduce UC symptoms by inhibiting inflammatory pathway activation, modulating the energy metabolism of intestinal epithelial cells, and protecting intestinal barrier function [56,57]. Research has shown that *Akkermansia* is capable of inhibiting the inflammatory responses of macrophages, thereby helping to reduce symptoms associated with UC [58]. Additionally, butyric acid encourages the polarization of macrophages towards the M2 subtype [59].

Various investigations have investigated the function of metabolites in sustaining intestinal homeostasis. To determine the metabolic pathways that may be linked to the anti-colitis effects of MFGMP, we analyzed the connection between metabolic pathways and UC. Notable modifications in amino acid metabolism were observed, involving changes in processes like the metabolism of tyrosine, the breakdown of lysine, and the biosynthesis of valine, leucine, and isoleucine. Among these metabolic pathways, tryptophan metabolism has garnered particular attention because of its role in intestinal homeostasis. Microbial derivatives of tryptophan can activate the aryl hydrocarbon receptor, thereby contributing to gut homeostasis [60]. This study observed a significant increase in tryptophan levels in the MP50 group compared to those in the DSS group. Additionally, the levels of metabolites, such as indole, indole-3-acetaldoxime, indole-3-acetonitrile, indole-3-acetamide, and indoleacetate, were elevated, along with an increase in the content of 5-hydroxy-indole acetate and 6-hydroxymelatonin.

The current body of evidence indicates that early-stage therapeutic interventions, such as dietary modifications and microbiota modulation, may effectively decelerate disease progression and mitigate morbidity in UC through multifaceted mechanisms [61,62]. Therefore, as both an immunomodulator and a modulator of the gut microbiota, MFGMP may offer prophylactic protection in healthy individuals, potentially lowering their risk of developing UC. Further studies are required to fully demonstrate the mechanism by which MFGMP components exert their anti-colitis activity. MFGMP can be incorporated into food as a functional ingredient for the prophylactic effects of UC. Many challenges must be overcome to realize the transition from laboratory to clinical applications, including establishing standardized large-scale industrial production and legal policies for MFGMP, as well as determining the optimal dosage.

## 5. Conclusions

The present study found that 50 mg/kg of MFGMP alleviated DSS-induced weight loss, reduced DAI in mice with UC, and significantly inhibited both systemic and local inflammation. Moreover, MFGMP significantly improved the dysregulated gut microbiota induced by UC, restoring both α-diversity and β-diversity and increasing probiotic *Lactobacillus* while decreasing the mucolytic bacteria *Akkermansia*. Metabolomic analysis suggested that MFGMP intervention affected metabolites, particularly amino acid metabolism. Specifically, L-valine was normalized, whereas the concentrations of L-leucine, L-lysine, and L-tyrosine increased almost twofold, and the concentration of L-tryptophan increased threefold. Furthermore, based on Spearman’s correlation analysis, increased amino acid levels were significantly negatively correlated with decreased pro-inflammatory cytokines in the colon, providing evidence that MFGMP alleviated intestinal inflammation by regulating amino acid metabolism. Lastly, the findings of this study revealed that MFGMP may regulate intestinal inflammatory processes through the Wnt/β-catenin signaling pathway. This finding further supports the potential of MFGMP as a functional food to prevent colonic inflammation.

## Data Availability

Data that support the findings of this study are available from the corresponding author upon reasonable request.

## Supplementary Materials

The following supporting information can be downloaded at: https://www.mdpi.com/article/10.3390/nu17050780/s1, Figure S1. SDS-PAGE profile of MFGMP. 1, 2, 3, and 4 are the MFGMP extracted at different times. Figure S2. (A) Weight change on day 7. (B) Length of the colon from each group. The MFGMP1 group was gavaged with only 25 mg/kg of MFGMP without DSS modeling, and the MFGMP2 group was gavaged with only 50 mg/kg of MFGMP without DSS modeling. Data were presented as means ± SD (n = 8). Statistical significance was determined using one-way ANOVA, followed by Tukey’s test. ns, non-significant. Table S1. Primer name. Table S2. Secondary difference metabolite.
