# Supplementary material for: Camel Milk Protein Ameliorates Ulcerative Colitis by Modulating Gut Microbiota and Amino Acid Metabolism
Source: Nutrients. 2025 Feb 24;17(5):780. doi: 10.3390/nu17050780 (PMC11902107; doi:10.3390/nu17050780)
Supplement: Supplementary file 1 [file nutrients-17-00780-s001.zip › nutrients-3428003-supplementary.pdf]

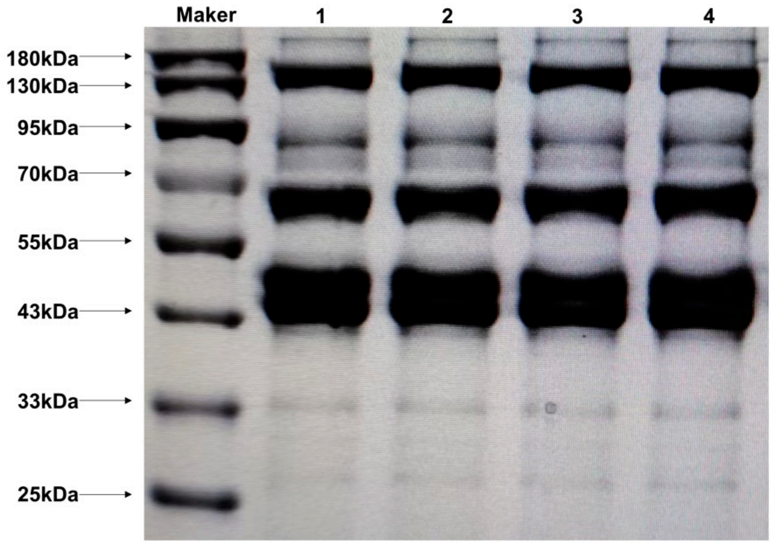

Fig.S1. SDS-PAGE profile of MFGMP. 1, 2, 3, and 4 are the MFGMP extracted at different times

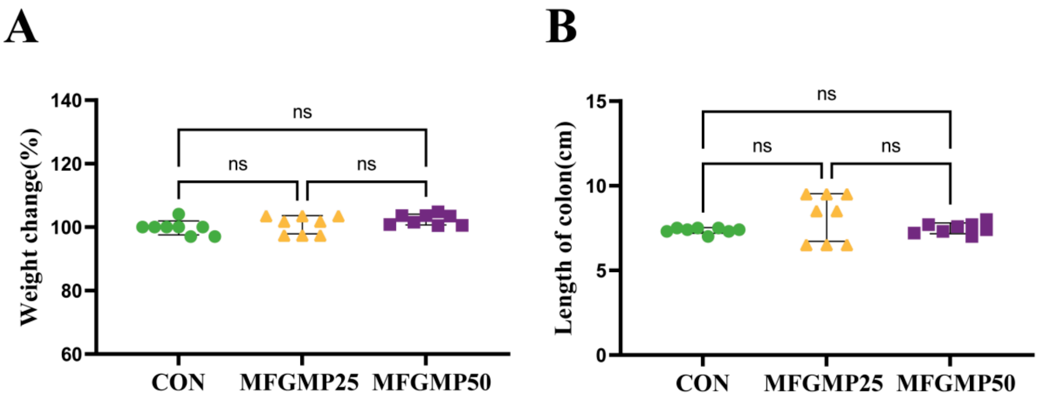

Fig.S2. (A) Weight change on day 7. **(B)** Length of the colon from each group. The MFGMP1 group was gavaged with only 25 mg/kg of MFGMP without DSS modeling, and the MFGMP2 group was gavaged with only 50 mg/kg of MFGMP without DSS modeling. Data were presented as means ± SD (n = 8). Statistical significance was determined using one-way ANOVA, followed by Tukey's test. ns, non-significant.

TABLE S1 Primer name

Primer name	Forward primer	Reverse primer
β-actin	CTGAGAGGGAAATCGTGCGT	CCACAGGATTCCATACCCAAGA
Jarid2	GGTCTGCTCAGGACTTACGG	GCACTGTTTGTACCTGCACC
Wnt-1	CTCTTTGGCCGAGAGTTCGT	TGCCTCGTTGTTGTGAAGGT
β-catenin	CGCTTGGCTGAACCATCACA	AGCAGCTTTATTAACTACCACCT
TNF-α	GGTGCCTATGTCTCAGCCTCTT	GCCATAGAACTGATGAGAGGGAG
Il-1β	GTCCGTCAACTTCAAAGAACAG	GCAAGTGTCTGAAGCAGCTAT
Il-6	TCCTTAGCCACTCCTTCTGT	AGCCAGAGTCCTTCAGAGA
IL-10	GAGGATCAGCAGGGGCCAGTAC	AAGGCAGTCCGCAGCTCTAGG
P50	TGGACAGCAAATCCGCCCTG	TGTTGTAATGAGTCGTCATCCT
P65	AGACCCAGGAGTGTTCACAGACC	GTCACCAGGCGAGTTATAGCTTCAG
I-kβ	CTGAAAGCTGGCTGTGATCCTGAG	CTGCGTCAAGACTGCTACACTGG

TABLE S2 Secondary difference metabolite

**Name**
p-Aminobenzoic acid
O-Phosphoethanolamine
2-(Methylamino)benzoic acid
4-Hydroxy-2-quinolone
7,8-Diaminononanoate
Sorbitol
O-Acetylcarnitine
4,4'-Dihydroxy-alpha-methylstilbene
N1,N12-Diacetylspermine
Ethylmorphine
Fluvoxamine
Lomefloxacin
Carboprost
3alpha,7alpha-Dihydroxy-12-oxo-5beta-cholanate
Tris(butoxyethyl)phosphate
Calcitriol
20a,22b-Dihydroxycholesterol
Coleonol
(R)-3-Hydroxybutyric acid
3-Methyl-2-oxovaleric acid
Oxoadipic acid
3,4-Dihydroxybenzeneacetic acid
gamma-Glutamylcysteine
(S,E)-Zearalenone
